# Specific antigen serologic tests in leprosy: implications for epidemiological surveillance of leprosy cases and household contacts

**DOI:** 10.1590/0074-02760160505

**Published:** 2017-09

**Authors:** Ana Paula Mendes Carvalho, Angélica da Conceição Oliveira Coelho, Rodrigo Correa-Oliveira, Francisco Carlos Félix Lana

**Affiliations:** 1 Universidade Federal de Minas Gerais, Escola de Enfermagem, Programa de Pós-Graduação em Enfermagem, Belo Horizonte, MG, Brasil; 2 Universidade Federal de Juiz de Fora, Faculdade de Enfermagem, Departamento de Enfermagem Básica, Juiz de Fora, MG, Brasil; 3 Fundação Oswaldo Cruz-Fiocruz, Centro de Pesquisas René Rachou, Laboratório de Imunologia Celular e Molecular, Belo Horizonte, MG, Brasil; 4 Universidade Federal de Minas Gerais, Escola de Enfermagem, Departamento de Enfermagem Materno-Infantil e Saúde Pública, Programa de Pós-Graduação em Enfermagem, Belo Horizonte, MG, Brasil

**Keywords:** leprosy, epidemiological monitoring, serologic tests, Mycobacterium leprae

## Abstract

**BACKGROUND:**

There is a lack of straightforward tests for field application and known biomarkers for predicting leprosy progression in infected individuals.

**OBJECTIVE:**

The aim was to analyse the response to infection by *Mycobacterium leprae* based on the reactivity of specific antigens: natural disaccharide linked to human serum albumin via an octyl (NDOHSA), a semisynthetic phenolic glycolipid-I (PGL-I); Leprosy Infectious Disease Research Institute Diagnostic-1 (LID-1) and natural disaccharide octyl - Leprosy Infectious Disease Research Institute Diagnostic-1 (NDOLID).

**METHODS:**

The study population consisted of 130 leprosy cases diagnosed between 2010 and 2015 and 277 household contacts. An enzyme-linked immunosorbent assay (ELISA) was used to analyse the reactivity of antibodies against NDOHSA, LID-1 and NDOLID. The samples and controls were tested in duplicate, and the antibody titer was expressed as an ELISA index. Data collection was made by home visits with application of questionnaire and dermatological evaluation of all household contacts to identify signs and symptoms of leprosy.

**FINDINGS:**

Significant differences in the median ELISA results were observed among leprosy cases in treatment, leprosy cases that had completed treatment and household contacts. Higher proportions of seropositivity were observed in leprosy cases in treatment. Seropositivity was also higher in multibacillary in relation to paucibacillary, with the difference reaching statistical significance. Lower titers were observed among cases with a longer treatment time or discharge. For household contacts, the differences according to the clinical characteristics of the leprosy index case were less pronounced than expected. Other factors, such as the endemicity of leprosy, exposure outside the residence and genetic characteristics, appeared to have a greater influence on the seropositivity.

**MAIN CONCLUSIONS:**

Serologic tests could be used as auxiliary tools for determining the operational classification, in addition to identifying infected individuals and as a strategy for surveillance of household contacts.

The clinical outcome after infection with *Mycobacterium leprae* is determined by cellular and humoral immunity (Bobosha et al. 2014). The manifestations of leprosy depend on such factors as the relationship between the etiological agent and the host, the bacterial load of the index case, exposure time (Douglas et al. 2004) and the socio-economic conditions of the exposed individual (Düppre et al. 2008).

The diagnosis of leprosy is mainly clinical and epidemiological and is achieved by analysis of the patient’s history, living conditions and dermatological and neurological evaluations (MS/SVS 2016). Laboratory tests such as histopathology, the Mitsuda reaction, slit skin smears and serology can be used to aid in the correct classification of patients when available. Laboratory tests are typically performed at reference centers and are not available in most health services (Contin et al. 2011).

Operational classification is used to define the treatment regimen with multidrug therapy according to the number of skin lesions. Patients with paucibacillary (PB) disease present with up to five lesions, whereas those with multibacillary (MB) disease present with more than five lesions. Additionally, a positive slit skin smear result and the presence of more than one compromised nerve result in classification the case as MB, regardless of the number of skin lesions (MS/SVS 2016).

A strategy for leprosy control in addition to early diagnosis and treatment involves the monitoring of household and social contacts through dermatological evaluation and vaccination with bacillus Calmette-Guérin (BCG) (MS/SVS 2016). Due to the long incubation period of *M. leprae* in the host, the general population should be kept under constant surveillance, particularly in endemic areas (de Souza et al. 2016).

The use of tests in addition to periodic evaluation through the clinical examination of contacts, including the details of their immune response and bacteriological status, may contribute to the identification of infected individuals and new leprosy cases. The identification of infected individuals may in turn contribute to early intervention and, thus, favorably affect disease control (Cardona-Castro et al. 2005).

For the containment of leprosy, it is necessary to establish biomarkers for the diagnosis and prognosis of an infection and its complications, such as reactional states (de Souza et al. 2016). An ideal test would allow the identification of individuals infected by *M. leprae* at risk of developing the disease or who contribute to the transmission of the bacillus. However, due to the difficulties of culturing *M. leprae* and the absence of a diagnostic gold standard, the development of a test that helps health professionals confirm the disease at an early stage among symptomatic patients and determine the appropriate treatment would be more feasible in the short term (Bahmanyar et al. 2016).

Despite the need to identify individuals infected with *M. leprae*, there is a lack of straightforward tests for field application and known biomarkers to predict disease progression in infected individuals. The currently applied tests include the widely investigated serologic tests to detect immunoglobulin M (IgM) against phenolic glycolipid-I (PGL-I, either native or semisynthetic) and the natural disaccharide linked to human serum albumin via octyl (NDOHSA), which is more useful for the identification of MB patients (Bobosha et al. 2014). The latter test has been used for patient classification, treatment monitoring and assessment of the risk of relapse as well as the selection of contacts at greater risk of becoming ill (Bührer-Sékula 2008, Moura et al. 2008).

In addition to native or semisynthetic PGL-I, other antigens that may have the potential to aid in the diagnosis of leprosy have been examined. The fusion protein Leprosy Infectious Disease Research Institute Diagnostic-1 (LID-1) was constructed from the ML0405 and ML2331 proteins, which were considered relevant diagnostic antigens after the analysis of a large panel of patient sera from MB patients from different regions in the world (Duthie et al. 2007).

The observation that some sera contain antibodies against one of the above antigens (PGL-1 and LID-1) but not the other supports the hypothesis that assessing the combination of the two antigens may increase the detection rate. Detailed analysis showed that although different magnitudes of response are observed for each antigenic component, the conjugate of these antigens, referred to as and natural disaccharide octyl - Leprosy Infectious Disease Research Institute Diagnostic-1 (NDOLID), reflects the average antibody reactivity to the individual antigens (Duthie et al. 2014a).

Given the above, the aim of the present study was to analyse the antibody response against *M. leprae* by testing for the reactivity to NDOHSA, LID-1 and NDOLID among leprosy cases and household contacts.

## SUBJECTS AND METHODS

*Study population* - The study population consisted of 130 leprosy cases diagnosed between 2010 and 2015 (45 were under treatment, and 85 had completed treatment) and 277 household contacts from urban areas of six municipalities in the north of Minas Gerais, Brazil, where the mean rate of new leprosy case detection was 41,31/100,000 inhabitants in the period from 2010 to 2015. In the household contacts group, we included individuals who resided in the same household as or near leprosy index cases diagnosed between 2010 and 2015 during a period of up to 5 years before the date of diagnosis, who were older than seven years of age. Individuals who were pregnant or suspected to be pregnant were excluded. Household contacts with a history of leprosy or who were contacts of more than one leprosy patient were also excluded because it would not have been possible to identify which case was responsible for the infection of the contact, and they may have been exposed to different clinical characteristics.

The majority of the leprosy cases were male (73.3% (n = 33) for leprosy cases in treatment and 57.6% (n = 49) for leprosy cases that had completed treatment). The average ages were 52 years (minimum: 15; maximum: 81) and 56 years (minimum: seven; maximum: 95) for leprosy cases in treatment and leprosy cases who had completed treatment, respectively. The clinical characteristics of the leprosy cases are presented in Table I. The average treatment time was eight months (minimum: less than one month; maximum: 17 months), and the average discharge time was 23 months (minimum: two months; maximum: 55 months). In the group of household contacts, the majority of subjects were female [55.2% (n = 153)], and the average age was 35 years (minimum: seven; maximum: 86).

**TABLE I:** Clinical characteristics of leprosy cases

Variables	Leprosy cases in treatment		Leprosy cases with completed treatment	
	
	n	(%)	n	(%)
Operational classification				
Paucibacillary	2	4.4	23	27.1
Multibacillary	42	93.3	62	72.9
Ignored	1	2.2	-	-
				
Clinical form of Madri				
Indeterminate	-	-	14	16.5
Tuberculoid	6	13.3	14	16.5
Borderline	26	57.8	37	43.5
Virchownian	9	20.0	18	21.2
Ignored	4	8.9	2	2.4
				
Slit skin smear status				
Negative	6	13.3	24	28.2
Positive	31	68.9	40	47.1
Ignored	8	17.8	21	24.7
				
Degree of disability at diagnosis				
Degree 0	20	44.4	31	36.5
Degree 1	18	40.0	43	50.6
Degree 2	3	6.7	7	8.2
Ignored	4	8.9	4	4.7

Total	45	100.0	85	100.0

*Data collection* - A database Sistema de Informação de Agravos de Notificação (SINAN) was used for the identification of leprosy cases and for information related to their clinical and epidemiological characteristics. Home visits were subsequently performed by researchers of the Studies and Research in Leprosy group (NEPHANS) of the Universidade Federal de Minas Gerais (UFMG), and a structured questionnaire was used to obtain information from the participants. A dermatological evaluation of all household contacts was conducted to identify signs and symptoms of leprosy. Individuals who presented with features suggestive of the disease were referred to the local health services for further diagnosis and treatment. Samples of blood were collected in two vacuum blood collection tubes containing clot activator and serum-separating gel for storage.

*Laboratory analyses* - An enzyme-linked immunosorbent assay (ELISA) was used to analyse the reactivity of antibodies against the selected antigens, NDOHSA, LID-1 and NDOLID, as described by Lobato et al. (2011) and Fabri et al. (2015). Antigens were provided by the Infectious Disease Research Institute (IDRI). The reconstitution and dilution of antigens were performed as indicated by IDRI. The samples and controls were tested in duplicate, and the antibody titer was expressed as an ELISA index (EI), where EI = sample optical density (OD) / cutoff OD. The cutoff value was calculated as the mean OD of the three negative controls plus three times the standard deviation of the value (Lobato et al. 2006, Fabri et al. 2015). EI values greater than 1.1 were considered positive (Lobato et al. 2006). The ELISA was repeated for samples that exhibited EI values classified as positive in a single well of a duplicate and samples whose EI values exhibited 25% or more variation between duplicates.

*Data analyses* - Epi Info version 3.5.1 software (Centers for Disease Control and Prevention, Atlanta, Georgia, United States of America) was used to prepare the database. GraphPad Prism software, version 5 (GraphPad Software, San Diego, California, United States of America) and Data Analysis and Statistical Software (STATA), version 11 (StataCorp, College Station, Texas, United States of America), were used for the statistical analyses. Descriptive analyses were performed to characterise anti-NDOHSA, LID 1 and NDOLID seropositivity in the studied groups. Spearman’s coefficient (*rho*) was used to test the strength of the correlations between the EI and the treatment time or discharge time of leprosy index cases, and the chi-squared test was used to analyse the proportion of seropositivity according to the operational classification of leprosy cases. McNemar’s test was employed to analyse the proportions of anti-NDOHSA, LID-1 and NDOLID seropositivity in each study group. The Kappa test was used to evaluate the concordance between the seropositivity results for each study group for different antigen combinations (NDOHSAxLID-1; NDOHSAxNDOLID; LID-1xNDOLID). The Kolmogorov-Smirnov test, histograms and normal charts were used to test data normality. The Mann-Whitney test (U) with Bonferroni correction was employed to compare the three evaluated groups. The statistical significance level was 5% (p ≤ 0.05).

*Ethics* - This study conformed to the Declaration of Helsinki and was reviewed and approved by the Research Ethics Committee of UFMG, Protocol number 13639.

## RESULTS

**Fig. 1:**
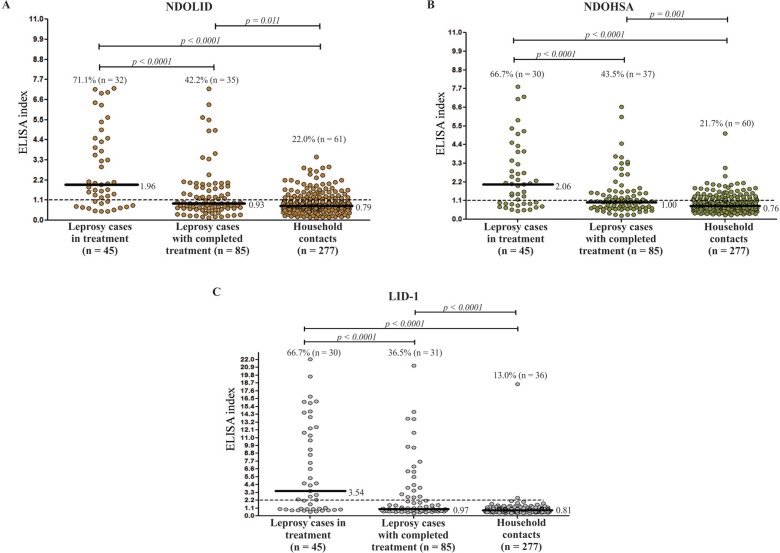
: anti-natural disaccharide linked to human serum albumin via octyl (anti-NDOHSA), anti-Leprosy Infectious Disease Research Institute Diagnostic-1 (anti-LID-1), and anti-natural disaccharide octyl - Leprosy Infectious Disease Research Institute Diagnostic-1 (anti-NDOLID) enzyme-linked immunosorbent assay (ELISA) indexes of leprosy cases according to treatment status and of household contacts. Each point in the figure corresponds to the ELISA index (EI) value of a participant. The median EI for each antigen is represented by a continuous horizontal line. The horizontal dashed line represents the positive cutoff (EI > 1.1). (A) NDOHSA. Mann-Whitney U-test with Bonferroni correction: leprosy cases in treatment versus leprosy cases that had completed treatment (p < 0.0001); leprosy cases in treatment versus household contacts (p < 0.0001); leprosy cases that had completed treatment versus household contacts (p = 0.011). (B) NDOLID. Mann-Whitney U-test with Bonferroni correction: leprosy cases in treatment versus leprosy cases that had completed treatment (p < 0.0001); leprosy cases in treatment versus household contacts (p < 0.0001); leprosy cases that had completed treatment versus household contacts (p = 0.001). (C) LID-1. Mann-Whitney U-test with Bonferroni correction: leprosy cases in treatment versus leprosy cases that had completed treatment (p < 0.0001); leprosy cases in treatment versus household contacts (p < 0.0001); leprosy cases that had completed treatment versus household contacts (p < 0.0001).

*Leprosy cases* - The analysis of seroreactivity to different antigens was performed based on the study groups described in the Subjects and Methods. Seropositivity to the three antigens (NDOHSA, LID-1 and NDOLID) was higher in the leprosy cases that were undergoing treatment (Fig. 1). The group of leprosy cases undergoing treatment showed the highest proportion of seropositivity to NDOHSA (71.1%; n = 32) and the same proportions of seropositivity to LID-1 and NDOLID (66.7%). For the leprosy cases that had completed treatment, a higher proportion of seropositivity was observed for NDOLID (43.5%, n = 37). The differences in the proportions of seropositivity were not significant for the leprosy cases in treatment (NDOHSA x LID-1: p = 0.687; NDOHSA x NDOLID: p = 0.625; LID-1 x NDOLID: p = 1.000) or for leprosy cases that had completed treatment (NDOHSA x LID-1: p = 0.481; NDOHSA x NDOLID: p = 0.774; LID-1 x NDOLID: p = 0.238).

Significant differences were observed in the median of EI values for anti-NDOHSA, LID-1 and NDOLID between the two groups of leprosy cases. The medians for the three evaluated antigens were higher in the group of leprosy cases in treatment, that presented higher LID-1 OD values. For the leprosy cases that had completed treatment, the medians were below the positive cutoff (Fig. 1).

In the group of leprosy cases in treatment, the Kappa test values for the comparison of antigens varied from 0.69 (substantial agreement) to 0.90 (near perfect agreement). Among the leprosy cases that completed treatment, the Kappa test values ranged from 0.56 (regular agreement) to 0.71 (substantial agreement). The values were statistically significant (Table II).

**TABLE II:** Concordance of anti-natural disaccharide linked to human serum albumin via octyl (anti-NDOHSA), anti-Leprosy Infectious Disease Research Institute Diagnostic-1 (anti-LID-1) and anti-natural disaccharide octyl - Leprosy Infectious Disease Research Institute Diagnostic-1 (anti-NDOLID) seropositivity by study group

Study group	*Kappa*	Valor p
Leprosy cases in treatment		
NDOHSA x LID-1	0.69	< 0.0001
NDOHSA x NDOLID	0.79	< 0.0001
NDOLID x LID-1	0.90	< 0.0001
		
Leprosy cases that had completed treatment		
NDOHSA x LID-1	0.56	< 0.0001
NDOHSA x NDOLID	0.71	< 0.0001
NDOLID x LID-1	0.56	< 0.0001
		
Household contacts		
NDOHSA x LID-1	0.17	0.0002
NDOHSA x NDOLID	0.63	< 0.0001
NDOLID x LID-1	0.13	0.0024

The proportion of seropositivity to at least one of the antigens evaluated was 75.6% (n = 34) for leprosy cases in treatment and 54.1% (n = 46) among leprosy cases that had completed treatment. The proportion of seropositivity to the three antigens evaluated simultaneously was 62.2% (n = 28) among cases in treatment and 25.9% (n = 22) among cases that had completed treatment.

The proportion of seropositivity was higher among MB cases than among PB cases, and this difference was statistically significant. Among MB cases, the proportion of positive anti-NDOHSA, LID-1 and NDOLID results was highly similar, while among PB patients, only one case exhibited anti-LID-1 seropositivity (Table III).

**TABLE III:** Anti-natural disaccharide linked to human serum albumin via octyl (anti-NDOHSA), anti-Leprosy Infectious Disease Research Institute Diagnostic-1 (anti-LID-1) and anti-natural disaccharide octyl - Leprosy Infectious Disease Research Institute Diagnostic-1 (anti-NDOLID) seropositivity of leprosy cases and household contacts according to the operational classification of leprosy index cases

Operational classification	Seropositivity^a^								

	NDOHSA			LID-1			NDOLID		
		
	n	(%)	p value^*^	n	(%)	p value^*^	n	(%)	p value^*^
Paucibacillary (PB) leprosy cases	5	18.5	<0.0001	1	3.7	<0.0001	4	14.8	<0.0001
Multibacillary (MB) leprosy cases	65	59.1		64	58.2		66	60.0	
Household contacts of PB leprosy cases	14	23.7	0.714	8	13.6	0.848	12	20.3	0.731
Household contacts of MB leprosy cases	46	21.5		27	12.6		48	22.4	

a: enzyme-linked immunosorbent assay (ELISA) index (EI) > 1.1; *: Chi-squared test.

The analysis of the treatment time of leprosy cases in treatment and the discharge time of leprosy cases that had completed treatment showed a negative correlation with the EIs for the three evaluated antigens. For the cases in treatment the three correlations were statistically significant and for leprosy cases that had completed treatment, only the antigen LID-1 presented a significant correlation (Table IV).

**TABLE IV:** Results of the Spearman test in different groups of individuals according to the enzyme-linked immunosorbent assay (ELISA) index (EI) for each antigen and the treatment time or discharge time

Treatment time or discharge time	NDOHSA		LID-1		NDOLID	
		
	*rho*	p value	*rho*	p value	*rho*	p value
Treatment time of leprosy cases in treatment	-0.356	0.016	-0.324	0.030	-0.336	0.024
Discharge time of leprosy cases that had completed treatment	-0.202	0.064	-0.312	0.004	-0.209	0.055

*rho*: spearman coefficient; LID-1: Leprosy Infectious Disease Research Institute Diagnostic-1; NDOHSA: natural disaccharide linked to human serum albumin via octyl; NDOLID: natural disaccharide octyl - Leprosy Infectious Disease Research Institute Diagnostic-1.

**Fig. 2:**
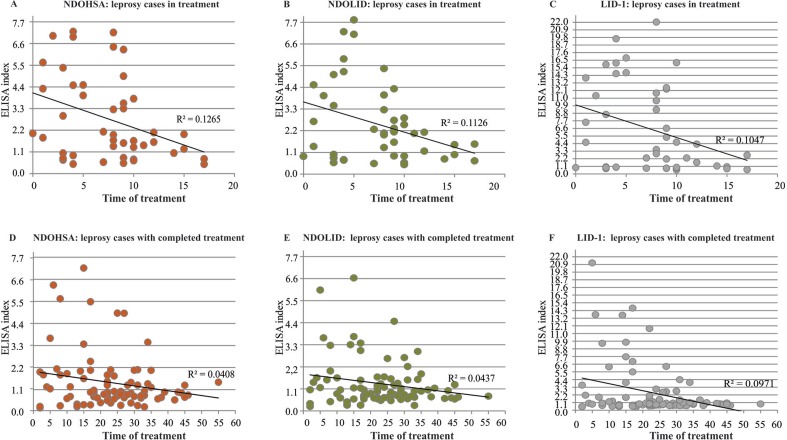
: anti-natural disaccharide linked to human serum albumin via octyl (anti-NDOHSA), anti-Leprosy Infectious Disease Research Institute Diagnostic-1 (anti-LID-1), and anti-natural disaccharide octyl - Leprosy Infectious Disease Research Institute Diagnostic-1 (anti-NDOLID) enzyme-linked immunosorbent assay (ELISA) indexes of leprosy cases according to treatment time or discharge time. Each point corresponds to the ELISA index (EI) values observed for a participant. The horizontal line represents the linear trend. (A) NDOHSA: leprosy cases in treatment; (B) NDOLID: leprosy cases in treatment; (C) LID-1: leprosy cases in treatment; (D) NDOHSA: leprosy cases that had completed treatment; (E) NDOLID: leprosy cases that had completed treatment; (F) LID-1: leprosy cases that had completed treatment.

Smaller EI values were observed among cases with longer treatment times or times to discharge. For the leprosy cases in treatment, the R^2^ values of the linear trends were greater than 10%, and the difference was more marked for NDOHSA (Fig. 2). The group of leprosy cases that had completed treatment was noteworthy in that the R^2^ values of the linear trends for NDOHSA and NDOLID were below 5%. The difference was more marked for LID-1; however, the value of R^2^ was below 10% (Fig. 2).

*Household contacts* - NDOHSA showed a higher proportion of seropositivity [22.0% (n = 61)] in the group of household contacts, and the proportion of anti-NDOLID seropositivity was very similar [21.7% (n = 60)]. Significant differences in the EIs for anti-NDOHSA, LID-1 and NDOLID were observed between the two groups of leprosy cases and the household contacts. For the household contacts and the leprosy cases that had completed treatment, the medians were below the positive cutoff (Fig. 1). The differences in the proportions of seropositivity were significant in the group of household contacts for NDOHSA x LID-1 (p = 0.003) and LID-1 x NDOLID (p = 0.006).

Kappa test values for the comparison of antigens varied from 0.13 (slight agreement) to 0.63 (substantial agreement). The values were statistically significant (Table II). The proportion of seropositivity to at least one of the evaluated antigens was 35.0% (n = 97), and the proportion of seropositivity to all three evaluated antigens simultaneously was 4.0% (n = 11).

According to the operational classification of leprosy index cases, the serological test of household contacts showed higher seropositivity only to NDOLID when we evaluated the contacts of MB cases compared to the contacts of PB cases. However, the observed differences were not statistically significant (Table III).

The analysis of the EIs of household contacts and leprosy cases showed a positive correlation for the three evaluated antigens (NDOHSA: *rho* = 0.058; LID-1: *rho* = 0.048; NDOLID: *rho* = 0.024). However, the differences were not statistically significant.

For household contacts, the treatment time or discharge treatment time of the leprosy cases did not show a significant correlation with the EIs for the three evaluated antigens (data not shown).

## DISCUSSION

The highest proportion of seropositivity for NDOLID was observed among leprosy cases that had completed treatment. For the other groups (leprosy cases in treatment and household contacts), NDOHSA showed the highest proportion of seropositivity. Only the differences in the proportions of NDOHSA versus LID-1 seropositivity and NDOLID versus LID-1 seropositivity among household contacts were significant. Similar results were observed in a study developed in China, in which the proportion of seropositivity to natural disaccharide linked to bovine serum albumin via octyl (NDOBSA) among contacts was higher than the proportion reported for NDOLID (Wen et al. 2014). However, the percentage of reactivity observed for NDOLID did not reflect the expected reactivity to NDOHSA and LID-1 (Duthie et al. 2014a). Furthermore, similar proportions of seropositivity were observed for the three evaluated antigens, particularly in the groups of leprosy cases.

The significant differences in the proportions of seropositivity observed for household contacts may be due to the fact that LID-1 can provide a clear diagnosis of infection with *M. leprae* before the appearance of signs that allow clinical diagnosis of leprosy (Duthie et al. 2007). Therefore, a lower proportion of contacts may present positive results. Furthermore, the similar proportions of anti-NDOHSA and NDOLID seropositivity could be related to the presence of the NDO portion of both antigens. For this reason, the differences in these antigens were not significant.

As expected, there was a higher proportion of seropositivity for the three antigens among MB cases than among PB cases.

The proportions of seropositivity observed for the three antigens in the group of MB cases (59.1% for NDOHSA; 58.2% for LID-1 and 60.0% for NDOLID) were lower than have been described in other studies for native or synthetic PGL-I (Duthie et al. 2007, Lobato et al. 2011, Araújo et al. 2012, Hungria et al. 2012, Wen et al. 2014, Fabri et al. 2015, Freitas et al. 2015), LID-1 (Duthie et al. 2007, Wen et al. 2014, Fabri et al. 2015, Freitas et al. 2015) and NDOLID (Wen et al. 2014, Fabri et al. 2015).

In the group of PB cases, the proportion of seropositivity for NDOHSA (18.5%) was higher than those observed in studies with native or synthetic PGL-I (Araújo et al. 2012, Hungria et al. 2012, Freitas et al. 2015). However, these proportions were lower than those observed in other studies with native or synthetic PGL-I (Duthie et al. 2007, Lobato et al. 2011, Wen et al. 2014, Fabri et al. 2015, Freitas et al. 2015) The anti-LID-1 (3.7%) and anti-NDOLID (14.8%) seropositivity rates were also lower than described in the literature for LID-1 (Duthie et al. 2007, Wen et al. 2014, Fabri et al. 2015, Freitas et al. 2015) and NDOLID (Wen et al. 2014, Fabri et al. 2015).

In these studies, the assessments were conducted in patients who were either untreated or at the beginning of treatment, with high bacterial loads. The differences in the treatment time of patients enrolled in these studies and the genetic characteristics of geographically distinct populations can influence the observed results. Moreover, failure in determining the operational classification by counting the number of skin lesions should be considered, which may cause under or over treatment. Comparison of the operational classification obtained by counting the number of skin lesions with the ML-Flow results in a study conducted in the state of Minas Gerais, Brazil showed that 29.8% of the patients may be insufficiently treated, and 17% may receive overtreatment if only the number of skin lesions is used as the criterion for classification (Grossi et al. 2008).

The differences in the proportions of seropositivity according to the operational classification were significant only for the leprosy cases group, which further supports the use of these antigens as a complementary tool for determining the operational classification and appropriate multidrug regimen.

The agreement indicated by the Kappa test and the proportion of seropositivity for the three antigens evaluated simultaneously was higher in the group of leprosy patients in treatment. This result suggests that patients with higher bacterial loads present higher reactivity to the evaluated antigens, as expected.

In the group of leprosy cases in treatment, LID-1 showed the highest EI values among the three analysed antigens. The highest median EI was also observed for this antigen. A similar result was described in another study, in which links that were considered strongly positive were also observed at higher frequencies for LID-1 and its components than for NDOBSA among MB patients (Duthie et al. 2008).

The observation of lower EI values between the cases with longer treatment times is consistent with the results of other studies that have identified a reduction in antibody titers during the treatment period for native or synthetic PGL-I, LID-1 and its individual components and NDOLID (Duthie et al. 2007, 2011, 2014b, Lobato et al. 2011, Rada et al. 2012, Freitas et al. 2015).

The decrease in antibody levels is related to a decreased bacterial load (Lobato et al. 2011). The decline is more evident among patients who presenting a higher bacillary index at the beginning of treatment (Duthie et al. 2014b). The less evident reduction of antibody levels in patients with a lower bacterial load is a potential reason for the less marked trend of reduction of EI values in cases that had completed treatment than in those that were in treatment.

A higher negative correlation between the EI and the time of discharge was observed for LID-1 and was significant for only this antigen. This result is similar to those described by other authors. There was a reduction of IgG antibodies specific for protein within three months after the beginning of treatment, and 10% reduction in anti-NDOHSA and a 30% reduction in the responses against proteins five months after the initiation of treatment (Duthie et al. 2011). A more robust decrease in the serologic responses against the proteins compared with PGL-I among MB patients in response to treatment has also been described (Freitas et al. 2015).

Regular assessment of antibody levels during treatment and after its completion has been suggested as an indicator for evaluating treatment efficacy (Duthie et al. 2007, 2011, Rada et al. 2012) and the need for additional treatment (Rada et al. 2012). Furthermore, screening for LID-1 antibodies in the general or at-risk population has been described as a possible method for accelerating the treatment of leprosy patients and, thus, affecting the transmission rate, as it would reduce the number of individuals who develop high bacterial loads (Duthie et al. 2007).

None of the variables analysed for leprosy index cases (operational classification, treatment time or discharge time and anti-NDOHSA, LID-1 and NDOLID seropositivity) had a significant influence on seropositivity in the group of household contacts. This result suggests that for the group of household contacts, other aspects, such as the endemicity of the municipality of residence, exposure to leprosy cases outside the residence and genetic characteristics may have a greater influence on seropositivity to the tested antigens than the clinical characteristics of leprosy index cases.
